# Spatial Orientation in a Cue–Target Task: Effects of Distance, Cue Direction, and Swimming Training Background

**DOI:** 10.3390/bs16071239

**Published:** 2026-07-21

**Authors:** Yunhao Zhang, Yi Liu, Yufei Ren, Senlin Lan, Ye Mao, Haoping Yang, Yi Peng

**Affiliations:** 1 School of Education, Beijing Sport University, Beijing 100084, China; 2025240665@bsu.edu.cn (Y.Z.); 2025240662@bsu.edu.cn (Y.L.); 2 School of Competitive Sports, Beijing Sport University, Beijing 100084, China; 2025240585@bsu.edu.cn (Y.R.); 2024240577@bsu.edu.cn (S.L.); 3 School of Psychology, Beijing Sport University, Beijing 100084, China; 18786494154@bsu.edu.cn

**Keywords:** visual-only condition, spatial orientation, spatial updating, cue direction, distance effect, swimming expertise

## Abstract

This study examined visually guided spatial updating and endpoint estimation under visual-only conditions and assessed whether performance varied by distance, cue direction, and swimming training background. Thirty-six right-handed male participants aged 18–20 years, including expert swimmers and amateur controls, completed a cue–target spatial orientation task with two distance conditions, short and long, and four cue directions, N, E, S, and W. Movement time, spatial error distance, and proportional movement time were analyzed using 2 × 2 × 4 mixed-design repeated-measures ANOVAs. Bayesian independent-samples comparisons were further conducted to quantify evidence for the null hypothesis of no robust group differences. The results indicated that task demands were the main determinants of performance. Compared with the short-distance condition, the long-distance condition yielded longer movement time, larger spatial errors, and higher proportional movement time, suggesting increased demands on spatial updating and movement execution. Cue direction also affected proportional movement time and spatial error distance, indicating direction-specific differences in visual cue integration and reference-frame transformation. By contrast, no robust overall group differences were found across the three primary behavioral measures, and the Bayesian analyses generally supported the null model. Although a significant Group × Distance interaction was observed for spatial error distance, this interaction reflected a group-related difference in the extent to which error increased from the short- to the long-distance condition, rather than evidence for a consistent expert advantage. Overall, performance in the present visual-only cue–target task was driven mainly by visuospatial updating load, attentional allocation, and task structure, whereas swimming-related expertise was not consistently expressed under the current task constraints.

## 1. Introduction

Spatial orientation enables individuals to represent their position in the environment, locate external targets, and guide behavior accordingly. It relies on multiple information sources, including vision, vestibular input, proprioception, and stored spatial representations. Previous research has shown that spatial orientation supports everyday navigation, direction judgment, position updating, and movement control in complex motor contexts (Wolbers & Hegarty, 2010; Chrastil & Warren, 2012). In the present study, spatial orientation was operationally defined in the context of a visual-only cue–target task as the ability to encode a visual direction cue and a landmark-referenced target relation, maintain this information during virtual navigation, update one’s position during movement, and estimate the target endpoint. Accordingly, the task was designed to assess visually guided spatial updating and endpoint estimation, rather than global spatial orientation ability.

Among the sensory systems involved in spatial orientation, vision is often regarded as one of the most direct and stable sources of information. Beyond static target recognition, vision contributes to external cue extraction, self-motion perception, spatial updating, and heading judgment. Classical studies of optic flow showed that individuals can derive self-motion direction from visual flow fields and use this information to judge heading and changes in spatial relations (Koenderink, 1986; Warren & Hannon, 1988; Warren et al., 1991). Lappe and Rauschecker (1994) further established the fundamental role of visual processing in heading perception. More recent work has extended this line of research from basic perceptual encoding to higher-level processes such as attentional allocation, working-memory maintenance, and multi-cue integration. Sun et al. (2023, 2024, 2025) showed that self-motion direction perception is shaped not only by immediate visual input but also by attentional resources and representational processing stages. Bayramova et al. (2021) similarly reported that, in immersive virtual environments, the contribution of vision to self-motion encoding becomes stronger when proprioceptive support and real-world landmark information are limited. Taken together, these findings indicate that studying spatial orientation under tightly controlled sensory conditions can help clarify the independent role of visual processing in spatial updating.

Spatial orientation, however, is not a unitary behavioral outcome. Rather, it is a dynamic process composed of multiple consecutive operations. Individuals must encode directional information and target location, preserve the relevant representation during movement or search, continuously update spatial information, and finally make a location judgment when confirming the endpoint. For this reason, spatial orientation performance should not be evaluated solely in terms of a single reaction-time or accuracy measure. A more informative approach is to assess multiple complementary dimensions, including execution efficiency, endpoint precision, and the allocation of processing time across stages. On this basis, the present study focused on movement time, spatial error distance (spatial error distance), and proportional time (proportional movement time) as the primary behavioral indicators. These variables were used to characterize execution efficiency, endpoint precision, and the proportion of total processing time allocated to the execution stage, respectively. Relative to a single-index approach, this multi-index framework is better suited to capturing the process-level characteristics of spatial orientation behavior under visual-only conditions.

A related question concerns whether long-term sport-specific experience enhances more general perceptual-cognitive processing. In sport cognition, this issue has remained central for many years. Meta-analyses and reviews suggest that athletes often outperform non-athletes in visual attention, perceptual judgment, response processing, and some spatial-cognitive tasks (Mann et al., 2007; Voss et al., 2010). Heppe et al. (2016) further proposed that sport expertise may be systematically related to visuospatial ability and basic cognitive ability. At the same time, the literature remains mixed with respect to transfer. Some findings support the view that prolonged sport training may promote relatively general perceptual-cognitive skills, whereas others indicate that athlete advantages depend heavily on the degree of correspondence between the experimental task and the athlete’s prior sport experience. This issue is particularly relevant in highly controlled laboratory tasks with neutral stimulus content, where any observed differences may reflect either generalized perceptual-cognitive transfer or task-specific processing strategies.

A more specific theoretical basis for expecting a possible expert advantage in the present study comes from research linking sport expertise to visuospatial processing, spatial working memory, and mental transformation abilities. Sport-specific training often requires repeated coordination between body orientation, directional control, and rapid updating of relative position, all of which may strengthen cognitive operations that are also relevant to spatial orientation performance in laboratory tasks. Heppe et al. (2016) argued that expertise in sport is systematically associated with visuospatial and basic cognitive skills. Similarly, Sato et al. (2022) reported that visuospatial working memory differs across sport types and may be stronger in athletes whose sports place greater demands on rapid spatial processing. In addition, Geisen et al. (2024) suggested that athletes from closed-skill sports may develop greater familiarity with the coupling of body rotation and spatial processing, which can support more efficient embodied mental-rotation performance. Related evidence from sport-related mental-rotation research also indicates that structured movement experience can selectively benefit spatial transformation processes (Feng et al., 2017). Taken together, these findings provide a theoretical rationale for expecting that swimmers with long-term systematic training may show an advantage in spatial orientation performance, particularly in tasks involving direction encoding, reference-frame transformation, and continuous updating of body-to-target relations. At the same time, whether such an advantage transfers to a highly controlled visual-only laboratory paradigm remains an open empirical question.

Swimming provides a particularly relevant context in which to test this possibility. Compared with many sports that rely on rich and continuously available external references, swimming is performed in an environment characterized by relatively limited visual reference information, continuous changes in body posture, and substantial demands on ongoing spatial updating. Swimmers must maintain stable judgments of lane direction and body orientation while continuously monitoring relative position during ongoing movement. Whether swimming-specific training influences the encoding and use of visuospatial cues therefore has clear theoretical relevance. Previous work suggests that swimming experience may be associated with time perception, rhythm processing, and some forms of perceptual judgment (Tobin & Grondin, 2012; Bove et al., 2017; Perrone et al., 2023). Water immersion has also been shown to influence vection and self-motion perception (Fauville et al., 2021). Although these studies do not directly address whether swimming-specific training alters spatial orientation performance under visual-only conditions, they provide a strong basis for examining visuospatial processing from the perspective of swimming expertise.

Despite this background, several limitations remain in the current literature. First, research on athlete perceptual-cognitive advantages has focused mainly on visual attention, time perception, or general response processing, whereas studies directly examining spatial orientation under visual-only conditions remain scarce. Second, many paradigms in navigation and spatial-updating research combine visual, vestibular, proprioceptive, and landmark information, which makes it difficult to isolate the independent contribution of visual cues to spatial orientation. Third, although athlete–non-athlete comparisons have been widely reported, it remains unclear whether any advantage associated with swimming expertise transfers to a neutral visuospatial task that minimizes overt sport-specific action semantics. Under these circumstances, a tightly controlled visual-only paradigm is useful not only for identifying the basic processing characteristics of spatial orientation but also for clarifying whether differences associated with training background can be observed when nonvisual sources of information are minimized.

Against this background, the present study employed a cue–target task under visual-only conditions to examine spatial orientation in participants with different swimming training backgrounds. The task included two distance conditions (short and long) and four cue directions (N, E, S, and W), and the primary behavioral indicators were movement time, spatial error distance, and proportional movement time. Using 2 × 2 × 4 mixed-design repeated-measures ANOVAs, we addressed two main questions. First, would distance and cue direction exert robust effects on spatial orientation performance under tightly controlled visual-only conditions? Second, would swimming training background be associated with differences in execution efficiency, endpoint precision, and time-allocation strategy? Based on the existing literature, we expected clear task-condition effects, particularly for distance and cue direction, because both factors are closely tied to spatial updating and directional transformation. Specifically, we expected the long-distance condition to result in longer movement time, larger spatial error distance, and higher proportional movement time than the short-distance condition, reflecting increased demands on spatial updating and movement execution. We also expected cue direction to influence task performance, because different directions may impose different demands on directional mapping and reference-frame transformation. Finally, we considered the possibility that participants with more extensive swimming training would show less performance decline under higher task demands, while recognizing that transfer from swimming expertise to a neutral laboratory task may depend on the degree of overlap between the task demands and the perceptual-motor demands of swimming.

## 2. Methods

### 2.1. Participants

Participant characteristics are presented in Table 1. Values are reported as mean ± standard deviation (M ± SD). World Aquatics Points (WA points) were used to index competitive swimming performance, with higher scores indicating higher performance level; this variable was reported only for the expert group, all of whom met the relevant national qualification standard. Swimming experience (years) referred to years of involvement in swimming, and exercise volume (hours/week) referred to weekly physical activity, defined as supervised swim training in the expert group and general physical activity in the amateur group, excluding any systematic swim training.

We used G*Power 3.1.9.7 (Faul et al., 2007) to estimate the minimum sample size required for a 2 (group) × 2 (dist_label) × 4 (cue_dir) mixed-design repeated-measures ANOVA. The analysis specified an effect size of *f* = 0.25 (Cohen, 1988), an alpha level of 0.05, statistical power of 0.95, a repeated-measures correlation of 0.50, and a nonsphericity correction of 1. The result indicated a minimum total sample size of 36 participants. This target sample size was broadly comparable to that used in previous studies comparing athletes and non-athletes.

Participant screening followed a two-stage procedure. In the first stage, all candidates completed a structured screening questionnaire covering demographic information, handedness, visual and auditory status, swimming training history, competition experience, weekly exercise volume, medical history, current medication use, and prior participation in highly similar experiments. In the second stage, conducted on the day of testing, the experimenter verified the questionnaire responses in person, confirmed the accuracy of group assignment, and recorded the demographic and background variables to be included in the final analysis, including age, handedness, visual and auditory status, years of systematic training, and weekly exercise volume. For the expert group, WA points and swimming background information were additionally verified.

The inclusion criteria applied to both groups were as follows: male sex; age between 18 and 20 years; right-handedness; normal or corrected-to-normal vision; normal hearing; and the ability to understand and complete the experimental task independently. To reduce variability related to sex, age, handedness, and motor-response characteristics, the sample was restricted to right-handed male participants aged 18–20 years. Although this restriction improved sample homogeneity and internal control, it also limited the generalizability of the findings. The exclusion criteria for both groups were as follows: self-reported neurological disorders, major psychiatric disorders, or a history of concussion or residual symptoms following traumatic brain injury; current use of medication that could affect central nervous system function; uncorrected visual or auditory impairment; prior participation in highly similar spatial orientation, navigation, or visual-judgment experiments; and failure to meet the predetermined task requirements during the practice phase or inability to complete the formal experiment independently. During post-experimental data screening, participants were also excluded if they showed excessive invalid trials, severe missing data in key conditions, or clear noncompliance with the task requirements.

Participants were included in the expert group if they were currently undergoing systematic swim training, had formal competition experience, had achieved at least the level of National First-Class Athlete, and showed a stable background of sport-specific training. These qualifications were verified against training and competition records. The amateur group was permitted to have limited recreational swimming experience, but such experience could not exceed 2 years. In addition, participants in this group could not have any history of systematic swim training, any competitive swimming experience, or any specialized athletic training comparable in intensity to that of the expert group within the past year.

The final analyzed sample comprised 36 male participants with a mean age of 19.23 ± 1.03 years. According to training background, participants were assigned to an expert group and an amateur group. The expert group included 20 participants, with a mean age of 19.09 ± 0.87 years, a mean of 10.36 ± 1.14 years of systematic swim training, a mean weekly training volume of 12.46 ± 3.37 h, and a mean primary-event WA points score of 695.09 ± 29.47. The amateur group included 16 participants, with a mean age of 19.36 ± 1.18 years, a mean swimming experience of 1.86 ± 0.77 years, and a mean weekly physical activity volume of 3.34 ± 1.54 h. Although participants in the amateur group could have had general exercise experience, none had received systematic swim training or had any competitive swimming experience. Detailed participant characteristics are shown in Table 2.

Before the experiment, all participants provided written informed consent. The study was approved by the Ethics Committee of Beijing Sport University (Approval No. 2024349H), and all procedures were conducted in accordance with the Declaration of Helsinki.

### 2.2. Apparatus and Stimuli

The experimental program was written in MATLAB 2021 and implemented using Psychtoolbox 3.1.7. Visual stimuli were presented on a 24-inch LCD monitor with a resolution of 1920 × 1080 pixels and a refresh rate of 60 Hz. Screen luminance was calibrated to approximately 100 cd/m^2^. Participants completed the task in a dim and relatively quiet room while seated approximately 60 cm from the monitor. To reduce the influence of circadian variation, all testing sessions were conducted between 09:00 and 12:00. Responses were collected using a standard keyboard. During cue confirmation and target navigation, participants used the keyboard to respond, with the arrow keys controlling forward, backward, leftward, and rightward movement, the space bar confirming the current position, and the ESC key terminating the experiment. To ensure consistency and reproducibility across participants, the same monitor and keyboard were used throughout data collection, and display parameters and response mappings were held constant.

The experiment was conducted under a visual-only condition, with all task information presented visually and no auditory stimuli delivered at any stage. No auditory stimuli were presented at any stage; all task information was delivered visually. The interface adopted a dark visual style. The screen background was set to near-black (RGB: 10, 10, 13), and the ground region was dark gray (RGB: 38, 38, 44). The scene included a starry background, ground texture, and left–right boundary markers to establish a relatively stable virtual environment. The core reference object was a tree, rendered in the three-dimensional scene with a trunk and canopy, and used as the primary landmark for the spatial-updating task. To ensure consistency across trials, the scene background, boundary settings, and landmark presentation were kept constant throughout the experiment.

The task stimuli comprised a CUE stage and a TARGET stage. During the CUE stage, a cue interface was presented at the center of the screen and divided into left and right regions. The left region displayed an upward arrow together with a direction letter indicating the cue direction for the current trial. Four cue directions were used: N, E, S, and W. The right region displayed a two-dimensional relative-position map, in which the landmark tree was shown as a green circle and the target was represented as a red triangle indicating its quadrant relative to the tree. Four target quadrants were used: NE, SE, SW, and NW. In this task, cue direction referred only to the direction letter displayed on the left side of the cue interface and did not indicate the location of the tree. The tree served as a fixed landmark, whereas the target quadrant indicated the target’s relative position with respect to the tree. Participants were therefore required to integrate the direction cue shown on the left with the landmark-referenced target relation shown on the right.

During the TARGET stage, participants entered the virtual scene and moved autonomously. The target itself was no longer visible, and participants had to complete spatial updating and position judgment on the basis of the information encoded during the earlier cue stage. To provide a stable directional reference throughout execution, an upward arrow and the letter “N” were continuously displayed in the upper-left corner of the screen without a background box. The position and orientation of this “N” arrow were fixed in screen coordinates throughout the TARGET stage. Specifically, the arrow always remained in the upper-left corner and always pointed upward on the screen; it did not rotate, shift, or otherwise change as a function of the participant’s movement direction or position in the virtual environment. Therefore, the “N” arrow served only as a constant visual reference for north, rather than as a dynamic cue indicating the participant’s current heading. Two distance conditions were used, short and long, corresponding to program parameter values of 32 and 64, respectively. The target location was not presented directly; instead, it was generated automatically by the program based on the tree location, cue direction, and target-quadrant relation, thereby ensuring standardized and consistent spatial relations across trials.

### 2.3. Experimental Task and Procedure

**Figure 1:**
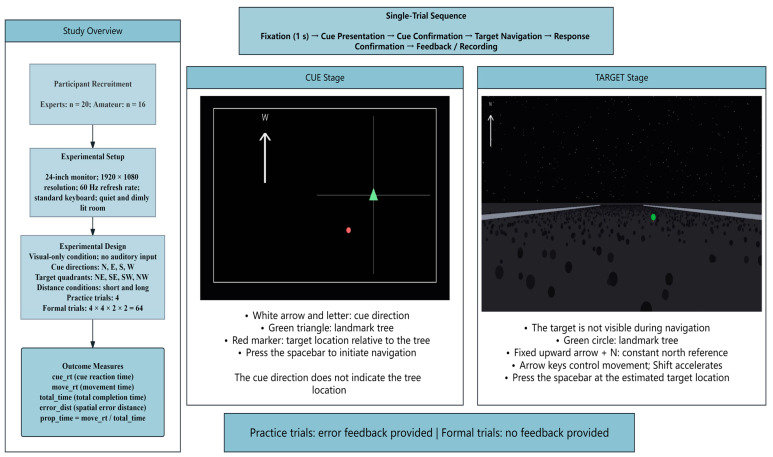
Study overview and single-trial sequence of the visual-only cue-target spatial orientation task. The study overview summarizes participant recruitment, the experimental setup, the experimental design, and the outcome measures. Each trial comprised fixation (1 s), cue presentation, cue confirmation, target navigation, response confirmation, and feedback/recording. During the CUE stage, the white arrow and accompanying letter indicated the cue direction, the green triangle represented the landmark tree, and the red circle represented the target location relative to the tree. During the TARGET stage, the target was not visible; the green circle represented the landmark tree, and the fixed upward arrow labeled “N” served as a constant north reference. Participants used the arrow keys to navigate and pressed the spacebar when they reached the estimated target location. Practice trials included error feedback, whereas formal trials did not.

The overall experimental procedure is illustrated in Figure 1. Participants first completed four practice trials with feedback, followed by 64 formal trials without feedback. Each trial began with a 1-s fixation, followed by cue presentation and confirmation, target navigation, response confirmation, and data recording.

### 2.4. Data Extraction and Statistical Analysis

The experimental program automatically recorded trial-level condition information, spatial-coordinate information, and behavioral measures and exported the data as CSV files. Recorded variables included participant number, run number, trial type, cue direction, target quadrant, distance label, planned distance, tree position, target position, final confirmed position, and timestamp.

A stage-based indicator framework was used to evaluate spatial orientation performance under visual-only conditions. The main behavioral measures included cue reaction time, movement time, total trial time, spatial error distance, and proportional time. Cue reaction time referred to the duration from cue onset to cue confirmation and was used to reflect processing efficiency during cue recognition, attentional allocation, and initial preparation. Movement time referred to the duration from the onset of the target search stage to the participant’s confirmation of the endpoint and was used to characterize the time cost of spatial execution and online correction. Total trial time referred to the total time from cue onset to final trial confirmation and was used to reflect overall task-completion efficiency. Spatial error distance was defined as the Euclidean distance between the participant’s final confirmed position and the program-generated target position and was used to reflect endpoint precision. Proportional time was defined as movement time/total_time and was used to capture the proportion of total processing time allocated to the execution stage, thereby reflecting time-allocation strategy between cue processing and execution-related correction. The present study focused on movement time, spatial error distance, and proportional movement time as the primary dependent variables. Together, these three indices represent execution efficiency, endpoint precision, and time-allocation strategy and therefore capture multiple aspects of spatial orientation performance under visual-only conditions.

During data preprocessing, the raw trial-level dataset was checked for field completeness and logical consistency, and trials with missing key variables, abnormal records, or clear noncompliance with the task requirements were removed. Data validity was further evaluated on the basis of experiment-operation quality indicators. At the participant level, each indicator was aggregated across distance conditions and cue directions. If missing values were present in a given condition, they were excluded from the denominator when computing the condition mean.

Statistical analysis was conducted using mixed-design repeated-measures ANOVAs. Group (group) was the between-subjects factor and included the amateur group and the expert group. Distance condition (distance condition) was a within-subjects factor with two levels, short and long, and cue direction (cue direction) was a within-subjects factor with four levels, N, E, S, and W. Separate 2 × 2 × 4 mixed-design repeated-measures ANOVA s were conducted for movement time, spatial error distance, and proportional movement time. For all main effects and interactions, F values, degrees of freedom, *p* values, and partial eta squared (*ηp*^2^) were reported. When the sphericity assumption was violated, Greenhouse–Geisser-corrected results were reported. When a main effect or interaction reached significance, simple-effects analyses or post hoc comparisons were conducted. To further evaluate whether the data supported the absence of robust group differences, Bayesian independent-samples comparisons were conducted on subject-level condition means for proportional movement time, spatial error distance, and movement time. Bayes factors were reported as BF_01_, indicating the relative evidence for the null model over the alternative model. BF_01_ values greater than 1 indicate evidence in favor of the null model, with values between 1 and 3 interpreted as anecdotal evidence and values between 3 and 10 interpreted as moderate evidence.

## 3. Results

### 3.1. Proportional Time (Proportional Movement Time)

**Figure 2:**
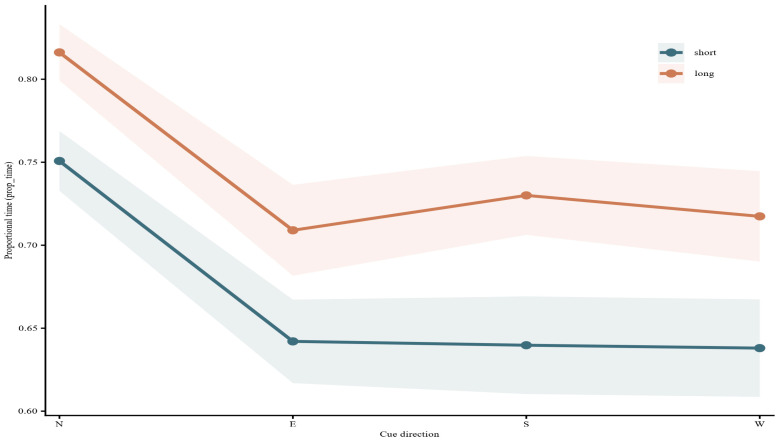
Proportional time across cue directions under short- and long-distance conditions. Mean proportional movement time values are plotted as a function of cue direction (N, E, S, and W) for the short- and long-distance conditions. Shaded bands indicate 95% confidence intervals. Higher values indicate a greater proportion of total trial time allocated to the execution stage.

A 2 × 2 × 4 mixed-design repeated-measures ANOVA was conducted for proportional movement time. The analysis revealed a significant main effect of distance condition, *F*(1, 34) = 98.749, *p* < 0.001, *ηp*^2^ = 0.744, indicating that the proportion of total trial time allocated to the execution stage was significantly higher in the long condition than in the short condition. In other words, under the longer distance condition, participants devoted a greater proportion of overall time to execution and online correction. The proportional movement time patterns across cue directions under the short- and long-distance conditions are shown in Figure 2.

The main effect of cue direction was also significant. Because the sphericity assumption for cue direction was violated (Mauchly’s W = 0.482, *p* < 0.001), Greenhouse–Geisser-corrected results were reported, *F*(1.978, 67.261) = 48.244, *p* < 0.001, *ηp*^2^ = 0.587, indicating significant differences in proportional movement time across cue directions. Within-subject trend analyses further showed significant linear, quadratic, and cubic trends for cue direction, *F*(1, 34) = 54.541, *p* < 0.001, *ηp*^2^ = 0.616; *F*(1, 34) = 51.386, *p* < 0.001, *ηp*^2^ = 0.602; and *F*(1, 34) = 26.291, *p* < 0.001, *ηp*^2^ = 0.436, respectively. These results indicate a clear systematic pattern in the distribution of proportional time across directions.

Descriptively, proportional movement time was relatively high in the N direction under the short condition. Under the long condition, proportional movement time increased across all directions, with the N direction remaining comparatively high. The main effect of group and all interaction effects were not significant (all *p*s > 0.05), indicating that the two groups showed broadly similar overall patterns of time-allocation strategy during the execution stage.

### 3.2. Spatial Error Distance (Spatial Error Distance)

**Figure 3:**
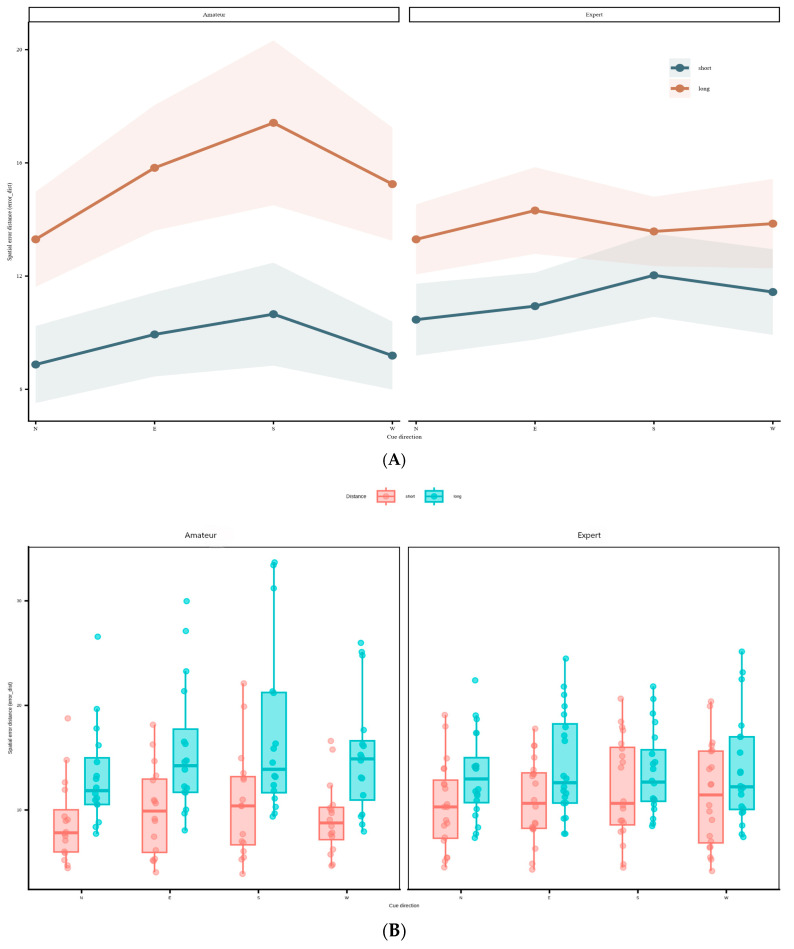
Spatial error distance across cue directions in the amateur and expert groups under short- and long-distance conditions. (**A**) Mean spatial error distance across cue directions (N, E, S, and W), shown separately for the amateur and expert groups. Lines represent the short- and long-distance conditions, and shaded bands indicate 95% confidence intervals. (**B**) Distribution of spatial error distance across cue directions in the amateur and expert groups. Boxplots show the median and interquartile range, and individual points represent participant-level values. Higher values indicate greater endpoint deviation from the target location.

A 2 × 2 × 4 mixed-design repeated-measures ANOVA was conducted on spatial error distance. The results showed a significant main effect of distance condition, *F*(1, 34) = 102.900, *p* < 0.001, *ηp*^2^ = 0.752, indicating that spatial error distance was significantly larger in the long condition than in the short condition. Longer distance therefore increased endpoint deviation. The main effect of cue direction was also significant. Because the sphericity assumption for cue direction was violated, Greenhouse–Geisser-corrected results were reported, *F*(2.337, 79.468) = 5.152, *p* = 0.005, *ηp*^2^ = 0.132, indicating significant differences in spatial error distance across cue directions. Thus, spatial error was not distributed uniformly across the four directions.The corresponding group-specific mean patterns and participant-level distributions are shown in Figure 3.

The main effect of group was not significant, *F*(1, 34) = 0.002, *p* = 0.963, *ηp*^2^ < 0.001, indicating that the amateur and expert groups did not differ in overall spatial-error level. In terms of interactions, the group × distance condition interaction was significant, *F*(1, 34) = 16.775, *p* < 0.001, *ηp*^2^ = 0.330, indicating that the magnitude of change in spatial error from short to long differed between groups. The group × cue direction interaction was not significant, *F*(3, 102) = 1.747, *p* = 0.162, *ηp*^2^ = 0.049. The distance condition × cue direction interaction was also not significant, *F*(3, 102) = 0.645, *p* = 0.588, *ηp*^2^ = 0.019. The three-way interaction among group, distance condition, and cue direction did not reach significance either, *F*(3, 102) = 2.476, *p* = 0.066, *ηp*^2^ = 0.068.

Simple-effects analyses showed that, in the amateur group, spatial error distance in the long condition was significantly larger than in the short condition, mean difference = −5.782, 95% CI [−7.186, −4.377], *p* < 0.001. In the expert group, spatial error distance was also significantly larger in the long condition than in the short condition, mean difference = −2.545, 95% CI [−3.532, −1.558], *p* < 0.001. Both groups therefore showed increased spatial error as distance increased, but the magnitude of the increase was larger in the amateur group.

Between-group comparisons within the same distance condition were not significant. Under the short condition, the mean difference between the amateur and expert groups was −1.551, 95% CI [−4.251, 1.149], *p* = 0.251. Under the long condition, the mean difference was 1.686, 95% CI [−1.872, 5.244], *p* = 0.339. The significant interaction therefore reflects different rates of increase from short to long across groups rather than a stable between-group difference within any single distance condition.

For spatial error distance, Bayesian comparisons also favored the null model, with BF_01_ values of 5.993 for the overall comparison, 3.001 for the short-distance condition, and 3.501 for the long-distance condition. These results indicate moderate evidence for the null model of no stable group difference. Accordingly, the significant Group × Distance interaction should be interpreted as a difference in the rate at which spatial error increased from the short- to the long-distance condition, rather than as evidence for a general expert advantage.

Descriptive statistics are presented in Table 3. Overall, mean spatial error distance values were higher in the long condition than in the short condition across all directions. The amateur group showed slightly higher means than the expert group in most conditions, but the overall between-group differences were small.

### 3.3. Movement Time

**Figure 4:**
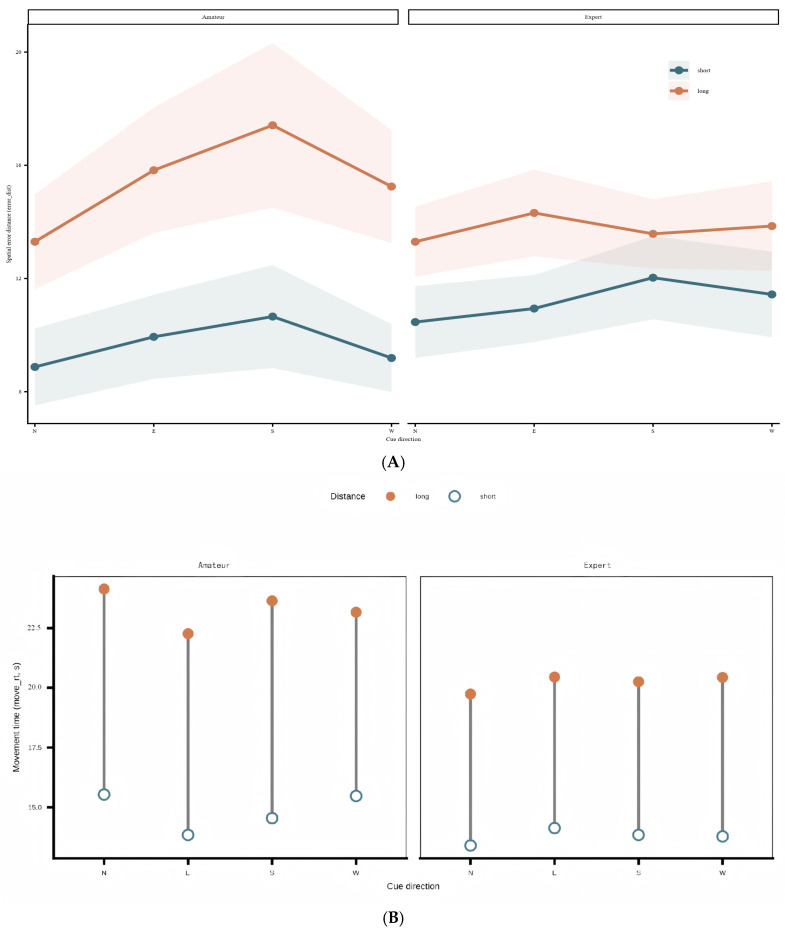
Movement time across cue directions in the amateur and expert groups under short- and long-distance conditions. (**A**) Mean movement time across cue directions (N, E, S, and W), shown separately for the amateur and expert groups. Lines represent the short- and long-distance conditions, and shaded bands indicate 95% confidence intervals. (**B**) Mean differences in movement time between the short- and long-distance conditions across cue directions in the amateur and expert groups. Open circles indicate the short-distance condition, filled circles indicate the long-distance condition, and line segments connect the two distance conditions within the same cue direction. Higher values indicate longer execution time during target navigation.

A 2 × 2 × 4 mixed-design repeated-measures ANOVA was conducted on movement time. The results showed a significant main effect of distance condition, *F*(1, 34) = 209.618, *p* < 0.001, *ηp*^2^ = 0.860, indicating that movement time was significantly longer in the long condition than in the short condition. Longer distance therefore required more time for task execution and online correction. The main effect of cue direction was not significant, *F*(3, 102) = 0.818, *p* = 0.487, *ηp*^2^ = 0.023, indicating that overall movement time did not differ significantly across directions. The main effect of group was also not significant, *F*(1, 34) = 1.049, *p* = 0.313, *ηp*^2^ = 0.030, indicating no overall difference in movement time between the amateur and expert groups. The group-specific movement-time patterns and the short-to-long differences across cue directions are shown in Figure 4.

In terms of interactions, the group × distance condition interaction did not reach significance, *F*(1, 34) = 3.926, *p* = 0.056, *ηp*^2^ = 0.104. The group × cue direction interaction was significant, *F*(3, 102) = 5.116, *p* = 0.002, *ηp*^2^ = 0.131, indicating that the pattern of movement-time change across cue directions differed between groups. Because the sphericity assumption for the distance condition × cue direction interaction was violated, Greenhouse–Geisser-corrected results were reported, *F*(2.434, 82.752) = 0.153, *p* = 0.893, *ηp*^2^ = 0.004, indicating that the interaction between distance and direction was not significant. The three-way interaction among group, distance condition, and cue direction was also not significant, *F*(2.434, 82.752) = 0.444, *p* = 0.682, *ηp*^2^ = 0.013.

Simple-effects analyses showed that movement-time differences between the amateur and expert groups did not reach significance at any cue direction. In the N direction, the mean difference between groups was 3.267, 95% CI [−0.892, 7.426], *p* = 0.119. In the E direction, the mean difference was 0.763, 95% CI [−3.253, 4.779], *p* = 0.701. In the S direction, the mean difference was 2.044, 95% CI [−2.366, 6.453], *p* = 0.352. In the W direction, the mean difference was 2.209, 95% CI [−2.214, 6.633], *p* = 0.316. Thus, the significant interaction did not take the form of a stable between-group difference in any single direction.

For movement time, Bayesian comparisons provided moderate evidence for the null model in the overall comparison and in the short-distance condition, with BF_01_ = 3.472 and BF_01_ = 4.913, respectively. The long-distance condition provided only anecdotal evidence for the null model, with BF_01_ = 2.548. Thus, the present data did not provide convincing evidence for a stable group difference in movement time.

Within-group pairwise comparisons showed that, in the amateur group, the uncorrected comparisons between N and E and between E and W reached significance. In the expert group, the uncorrected comparison between N and E also reached significance. After Bonferroni correction, however, none of these pairwise differences remained significant. Accordingly, the significant group × cue direction interaction should be interpreted cautiously, as it appears to reflect differences in the overall directional profile rather than robust pairwise differences at specific directions.

From the descriptive mean pattern, movement time values were lower across directions in the short condition and increased overall in the long condition. In general, the amateur group showed slightly higher means than the expert group in most conditions, but the overall between-group differences were small. Although the overall main effect of cue direction was not significant, the two groups did not show identical fluctuation patterns across directions, which is consistent with the significant group × cue direction interaction.

Descriptive statistics are presented in Table 4. Overall, movement time values were higher in the long condition than in the short condition across all directions, and the amateur group tended to show slightly higher mean values than the expert group in most conditions, although the overall between-group differences were small.

### 3.4. Bayesian Evidence Regarding Group Differences

To further evaluate the absence of robust group differences, Bayesian independent-samples comparisons were conducted on subject-level condition means. As shown in Table 5, most BF_01_ values favored the null model over the alternative model. For proportional movement time and spatial error distance, the BF_01_ values consistently indicated moderate evidence for the null model. For movement time, the overall and short-distance comparisons also provided moderate evidence for the null model, whereas the long-distance comparison provided only anecdotal evidence. These findings suggest that the present data did not provide convincing evidence for a stable group difference between expert swimmers and amateur controls.

## 4. Discussion

The present study examined visually guided spatial updating and endpoint estimation in a cue–target task under visual-only conditions and compared behavioral performance between participants with different swimming training backgrounds. The findings indicate that spatial orientation performance was influenced primarily by task conditions, with distance and direction emerging as the most prominent determinants, whereas stable group differences did not appear. This pattern suggests that, when nonvisual information is strictly restricted, spatial orientation performance is driven more directly by visuospatial processing load and task demands. By contrast, any advantage that might arise from swimming-specific training did not manifest as a robust overall effect in the present experimental paradigm. The current findings are therefore more appropriately interpreted as evidence for the dominant role of task conditions in shaping spatial orientation performance than as evidence for a broad sport-specific advantage.

### 4.1. Spatial Orientation Performance Under Visual-Only Conditions Was Driven Primarily by Task Conditions

The present study showed that distance condition had a clear influence on spatial orientation performance. Compared with the short condition, the long condition was associated with longer movement time, larger endpoint error, and higher proportional movement time. This pattern suggests that increasing target distance increased the demands of spatial updating under visual-only conditions.

In the present task, participants had to remember the target relation, monitor their position during movement, and update the relation between their current position and the estimated target location. When the distance was longer, this process became more demanding. Participants needed to maintain the spatial representation for a longer period and rely more on visual information to guide movement and correct errors. Because vestibular and proprioceptive cues were minimized, visual monitoring and attentional control became especially important.

The larger endpoint error and longer movement time in the long condition may therefore reflect the accumulation of small errors during navigation. As movement distance increased, inaccuracies in direction mapping and position updating may have gradually increased, leading to poorer endpoint precision and longer execution time. The increase in proportional movement time further suggests that participants spent a greater share of total task time on movement-related monitoring and correction after the initial cue-encoding stage.

Therefore, distance should not be interpreted only as a physical increase in travel length. Rather, it also increased the cognitive demands of maintaining, updating, and correcting spatial representations. Under visual-only conditions, these demands appeared to shape performance more strongly than swimming training background.

### 4.2. Significant Direction Effects Suggest Direction-Specific Processing Differences in Spatial Orientation

The present study also showed significant performance differences across cue directions. This finding indicates that spatial orientation under visual-only conditions was not equivalent across all directions. Although the four directional conditions were formally symmetrical in the design, they may have required different cognitive operations during task performance.

In the present task, cue direction was not merely a visual label. Participants had to integrate the cue direction shown on the left side of the screen with the landmark-referenced target relation shown on the right side. This process likely required attentional shifting between the two cue regions, transformation of the visual direction into an internal spatial reference frame, and maintenance of the transformed target relation during navigation. Therefore, differences across N, E, S, and W conditions may reflect variation in directional mapping, attentional allocation, and reference-frame transformation demands.

This interpretation is consistent with previous studies showing that body orientation and spatial transformation influence performance in virtual navigation and mental-rotation tasks. Moon et al. (2023) reported that body orientation affects navigation experience and behavioral performance in virtual environments. Feng et al. (2017) showed that sport expertise can selectively influence different stages of mental rotation, and Geisen et al. (2024) further linked embodied mental-rotation ability with the type of motor skill. In the present study, these findings suggest that cue direction may have changed the amount of cognitive effort required for visual reference transformation and spatial updating.

At the same time, the present results should be interpreted cautiously. The direction effect should not be attributed to a single mechanism such as mental rotation or directional preference alone. A more appropriate interpretation is that cue direction shaped performance by changing the difficulty of integrating visual cues, transforming spatial references, and updating the remembered target location. Thus, the significant direction effect provides evidence for direction-specific variation in spatial processing rather than direct evidence for one fixed cognitive mechanism.

### 4.3. Possible Reasons Why Stable Group Differences Did Not Emerge Across Training Backgrounds

A central finding of the present study was that robust overall group differences were not observed between participants with different swimming training backgrounds. Under the current task paradigm, the expert group did not show a clear overall advantage in movement time, spatial error distance, or proportional movement time.

The Bayesian analyses provide additional support for this cautious interpretation. Across proportional movement time, spatial error distance, and movement time, most BF_01_ values favored the null model, suggesting that the current data were more consistent with no stable group difference than with a reliable expert-advantage model. However, this finding should not be interpreted as definitive evidence that swimming expertise has no influence on spatial orientation. Rather, it indicates that the present visual-only cue–target task did not provide convincing evidence for stable expertise-related advantages.

This result can be understood from the perspective of sport skill transfer and task specificity. Previous studies have linked sport expertise to visuospatial ability, spatial working memory, and mental transformation processes (Heppe et al., 2016; Sato et al., 2022). Research on embodied mental rotation has also suggested that structured movement experience may support the coupling between body orientation and spatial processing (Geisen et al., 2024; Feng et al., 2017). On this basis, it was theoretically reasonable to expect that swimmers with long-term systematic training might show advantages in a task involving direction encoding, reference-frame transformation, and spatial updating.

However, the present task may be closer to a far-transfer context than to a near-transfer context. Real swimming requires continuous control of body orientation, rhythm, lane direction, and body–environment relations in an aquatic setting. These processes are strongly embodied and usually depend on the combined use of visual, vestibular, proprioceptive, tactile, and aquatic environmental cues. In contrast, the present task required keyboard-based navigation in a highly controlled visual environment, without body rotation, water immersion, or sport-specific movement demands. This limited perceptual–motor overlap may help explain why stable group differences did not emerge.

The visual-only design may therefore have reduced the likelihood that swimming-specific spatial advantages would be expressed and made performance depend more on general visuospatial updating and endpoint estimation. This interpretation is consistent with evidence that water immersion can alter self-motion perception (Fauville et al., 2021) and that athlete advantages in perceptual-cognitive tasks are often task-dependent rather than universally expressed (Imperiali et al., 2025). It should also be noted that the present sample size and task-difficulty range may have limited the detection of group differences. If both groups were mainly challenged by the same basic spatial-updating demands, distance and cue direction would naturally emerge as the dominant sources of variation, whereas training-related differences could remain statistically undetectable.

### 4.4. Limitations and Future Directions

Several limitations should be acknowledged. First, the present experiment employed only a visual-only condition. Although this design greatly improved internal control and allowed the independent contribution of visual processing to be examined more precisely, it still differs substantially from the multisensory spatial orientation process involved in real swimming. In actual swimming, spatial orientation depends on the joint contribution of vision, proprioception, vestibular information, and aquatic environmental cues. The single-cue design used here cannot fully reproduce that real-world process. In addition, all tasks were completed in a laboratory setting, which limits ecological validity and makes it difficult to generalize the findings directly to spatial orientation performance in real swimming. Laboratory environments offer strong experimental control, but they lack crucial features of the aquatic setting and bodily movement state. Finally, the present analyses relied primarily on behavioral measures. Although movement time, spatial error distance, and proportional movement time provide useful information, they are insufficient to fully reveal the mechanisms underlying the distance and direction effects or to clarify the internal processing dynamics of spatial updating and directional transformation.

The relatively homogeneous sample also limits the generalizability of the findings. All participants were right-handed male university students aged 18–20 years. Although this design improved experimental control, it precluded examination of sex-related differences in visuospatial processing, age-related variation in spatial updating, and the influence of different training or educational environments. Future studies should include more diverse samples to determine whether the present findings can be generalized beyond young male university students.

Future research could extend the present work in several ways. Eye tracking, EEG, and more immersive virtual-reality navigation paradigms could be incorporated to examine the process of spatial updating and directional transformation in greater detail and to identify the underlying cognitive mechanisms more directly. In addition, future studies could increase task complexity, introduce multisensory cues such as proprioceptive and vestibular information, and expand the hierarchy of task demands in order to test whether individuals with different training backgrounds show more stable spatial orientation advantages under higher cognitive load or under conditions more closely resembling actual swimming. Such work would help clarify more comprehensively how swimming-specific training influences spatial orientation ability.

## 5. Conclusions

Using a cue–target task under visual-only conditions, the present study examined visually guided spatial updating and endpoint estimation in participants with different swimming training backgrounds. The findings indicate that performance was shaped mainly by task demands, particularly distance and cue direction, whereas stable training-background effects were not observed under the present task constraints. Bayesian analyses provided additional support for the null model of no robust group differences, suggesting that expertise-related advantages were not consistently expressed in this visual-only paradigm. Overall, performance in the current task appeared to depend more on visuospatial updating load, attentional allocation, and reference-frame transformation than on swimming training background itself. Therefore, although the controlled design helps clarify spatial performance under visual-only conditions, the findings should not be generalized directly to real swimming environments. Future studies should adopt more immersive, multisensory, neurophysiological, and sport-specific paradigms to examine whether swimming expertise influences spatial orientation when visual, vestibular, proprioceptive, and aquatic environmental information are jointly available.

## Figures and Tables

**Table 1:** Participant characteristics.

Group	Age	Sex	Experience (Years)	Exercise Volume (Hours/Week)	WA Points
Expert group	19.09 ± 0.87	20 male	10.36 ± 1.14	12.46 ± 3.37	695.09 ± 29.47
Amateur group	19.36 ± 1.18	16 male	1.86 ± 0.77	3.34 ± 1.54	-
Total	19.23 ± 1.03	36 male	-	-	-

**Table 2:** Descriptive statistics for proportional movement time across distance and cue directions (M ± SD).

Distance Condition	Cue Direction	Amateur Group (n = 16)	Expert Group (n = 20)	Total (N = 36)
short	N	0.7462 ± 0.08434	0.7544 ± 0.08200	0.7508 ± 0.08194
short	E	0.6496 ± 0.13474	0.6361 ± 0.09862	0.6421 ± 0.11449
short	S	0.6454 ± 0.13362	0.6352 ± 0.13725	0.6397 ± 0.13381
short	W	0.6466 ± 0.14915	0.6311 ± 0.12344	0.6380 ± 0.13367
long	N	0.8164 ± 0.09191	0.8160 ± 0.06528	0.8162 ± 0.07703
long	E	0.7260 ± 0.13812	0.6954 ± 0.11422	0.7090 ± 0.12449
long	S	0.7401 ± 0.10804	0.7220 ± 0.11016	0.7300 ± 0.10804
long	W	0.7379 ± 0.13363	0.7010 ± 0.11606	0.7174 ± 0.12374

**Table 3:** Descriptive statistics for spatial error distance across distance and cue directions (M ± SD).

Distance Condition	Cue Direction	Amateur Group (n = 16)	Expert Group (n = 20)	Total (N = 36)
short	N	8.876 ± 3.928	10.461 ± 4.170	9.757 ± 4.085
short	E	9.939 ± 4.288	10.938 ± 3.893	10.494 ± 4.045
short	S	10.655 ± 5.251	12.029 ± 4.829	11.418 ± 4.996
short	W	9.191 ± 3.464	11.437 ± 4.979	10.439 ± 4.459
long	N	13.299 ± 4.846	13.298 ± 4.065	13.299 ± 4.363
long	E	15.825 ± 6.397	14.316 ± 5.032	14.987 ± 5.644
long	S	17.414 ± 8.402	13.578 ± 4.027	15.283 ± 6.542
long	W	15.250 ± 5.770	13.853 ± 5.189	14.474 ± 5.420

**Table 4:** Descriptive statistics for movement time across distance and cue directions (M ± SD).

Distance Condition	Cue Direction	Amateur Group (n = 16)	Expert Group (n = 20)	Total (N = 36)
short	N	15.533 ± 5.562	13.396 ± 5.008	14.346 ± 5.294
short	E	13.840 ± 4.688	14.126 ± 5.677	13.999 ± 5.190
short	S	14.541 ± 6.329	13.841 ± 5.473	14.152 ± 5.793
short	W	15.471 ± 5.331	13.777 ± 5.050	14.530 ± 5.172
long	N	24.137 ± 7.937	19.739 ± 6.496	21.693 ± 7.404
long	E	22.265 ± 7.552	20.452 ± 6.431	21.258 ± 6.908
long	S	23.641 ± 8.010	20.254 ± 7.080	21.759 ± 7.591
long	W	23.163 ± 8.991	20.438 ± 6.929	21.649 ± 7.912

**Table 5:** Bayesian evidence for group differences across dependent variables.

Dependent Variable	Comparison	BF_01_	Evidence
Proportional movement time	Overall	5.443	Moderate evidence for the null model
Proportional movement time	Short distance	5.858	Moderate evidence for the null model
Proportional movement time	Long distance	4.816	Moderate evidence for the null model
Spatial error distance	Overall	5.993	Moderate evidence for the null model
Spatial error distance	Short distance	3.001	Moderate evidence for the null model
Spatial error distance	Long distance	3.501	Moderate evidence for the null model
Movement time	Overall	3.472	Moderate evidence for the null model
Movement time	Short distance	4.913	Moderate evidence for the null model
Movement time	Long distance	2.548	Anecdotal evidence for the null model

Note: BF_01_ indicates the Bayes factor in favor of the null model over the alternative model. Values between 1 and 3 were interpreted as anecdotal evidence, whereas values between 3 and 10 were interpreted as moderate evidence for the null model.

## Data Availability

The de-identified behavioral data and analysis scripts supporting the findings of this study are available from the corresponding author upon reasonable request.
